# Pulmonary function, diffusing capacity, and forced oscillometry after recovery from COVID-19 in young, healthy, recreationally active men and women

**DOI:** 10.1016/j.ijregi.2025.100668

**Published:** 2025-05-11

**Authors:** Mohini Bryant-Ekstrand, Peter Luu, Thomas Gooding, Rachel Jaten, Andrew Thomas Lovering, Hans C. Haverkamp

**Affiliations:** 1 Department of Human Physiology, University of Oregon, Eugene, USA; 2 Washington State University-Health Sciences Spokane, College of Medicine, Department of Nutrition & Exercise Physiology, Spokane, USA

**Keywords:** COVID19, Forced oscillation testing, Lung diffusing capacity, Lung function tests, Spirometry

## Abstract

**Objectives:**

This study aimed to examine the long-term impact of COVID-19 on pulmonary function (spirometry and forced oscillometry technique, and lung diffusing capacity for carbon monoxide [DL_CO_]) in a large group of young, recreationally active adults.

**Methods:**

A total of 71 participants (aged 21 years) who had recovered from a positive COVID-19 infection (COVID^+^) and 55 participants (aged 24 years) who had never tested positive for COVID-19 (COVID^−^) performed routine spirometry and DL_CO_. Some patients also completed forced oscillometry technique (*n* = 22 COVID^+^; *n* = 18 COVID^−^).

**Results:**

The ratio of forced expiratory volume in 1 second to forced vital capacity (FEV_1_/FVC) and forced expiratory flow between 25% and 75% of FVC (FEF_25-75%_) were slightly but significantly lower in patients who were COVID^+^ than those who were COVID^−^ (FEV_1_/FVC = 93.6 ± 8.3 vs 96.9 ± 6.7%-predicted, *P* = 0.019; FEF_25-75%_ = 86.0 ± 21.2 vs 94.5 ± 21.1%-predicted, *P* = 0.015, respectively). There were no associations between any pulmonary function variables or DL_CO_ and the number of days between COVID-19 infection and testing.

**Conclusions:**

Our findings provide evidence that pulmonary function and DL_CO_ are largely preserved after recovery from mild COVID-19 in young, healthy, active men and women.

## Highlights

• Spirometry is preserved after recovery from COVID-19 virus in young, healthy adults.
• Lung diffusing capacity for carbon monoxide is normal after recovery from COVID-19.
• Lung mechanics via oscillometry are normal after recovery from COVID-19.

## Introduction

Adults aged 18-29 years account for the greatest number of COVID-19 cases in the United States than any other age group, reaching nearly 20 million in August 2023 (20.1% of all cases) [1]. Findings from examinations of pulmonary function after COVID-19 in this demographic are equivocal, with reports of normal to mildly impaired spirometry and lung diffusing capacity (DL_CO_) in the weeks to several months after COVID-19 infection [[2], [3], [4], [5], [6]]. Small patient numbers, variable or unknown timespan between symptomatic infection and testing, lack of control groups, and differences in vaccination status make it difficult to synthesize the studies. In addition, there are no publications reporting respiratory oscillation mechanics after COVID-19. Forced oscillation testing provides a detailed assessment of respiratory mechanics that complements findings gleaned from traditional spirometry.

We assessed spirometry, forced oscillometry, and DL_CO_ in young, healthy individuals after recovery from COVID-19 and in healthy controls never testing positive for COVID-19. We hypothesized that spirometric measures of forced expiratory airflow, oscillometric measures of airway function, and DL_CO_ would be slightly impaired in patients with a previous COVID-19 diagnosis.

## Material and methods

This study received approval from the University of Oregon Research Compliance Services. All studies were performed in accordance with the 2013 Declaration of Helsinki. A total of 71 patients who had recovered from a positive COVID-19 infection (COVID^+^) and 55 persons never testing positive for COVID-19 (COVID^−^) responded to flyers posted in Eugene, OR, USA and Spokane, WA, USA. Patients completed the MESA COVID-19 history questionnaire [7]. Participants who were COVID^+^ were classified as asymptomatic, mild, or moderate in accordance with modified National Institute of Health standards. Asymptomatic was defined as individuals with a positive SARS-CoV-2 test but no symptoms. Mild illness was defined as individuals with any signs and/or symptoms of COVID-19 but no “shortness of breath, dyspnea, or abnormal chest imaging.” Moderate illness was defined as individuals with lower respiratory disease but with pulse oximetry ≥94%.

All participants completed one laboratory visit between December 2021 and August 2022. Spirometry and DL_CO_ were performed according to American Thoracic Society/European Respiratory Society standards. Predicted values and lower limits of normal (LLN) were from Global Lung Initiative for spirometry [8] and DL_CO_ [9]. Forced oscillation testing (Thorasys Tremoflow) was conducted on participants tested at Washington State University (*n* = 22 COVID^+^; *n* = 18 COVID^−^). Outcome measures included respiratory resistance (R_5_), frequency dependence of resistance (R_5-20_), reactance (X_5_), and area of reactance between X_5_ and the resonant frequency (AX) according to current European Respiratory Society guidelines.

Unpaired Student’s *t*-tests were used to compare percentage-predicted forced vital capacity (FVC), forced expiratory volume in 1 second (FEV_1_), FEV_1_/FVC, and DL_CO_ between COVID^−^ and COVID^+^. The Mann–Whitney test was used to compare forced expiratory flow between 25% and 75% of FVC (FEF_25-75%_) and the oscillometric outcomes between groups because the data were not normally distributed. Associations between variables were determined by Pearson’s product moment correlation and chi-square analyses. Statistical significance was set at *P* <0.05.

## Results

Table 1 presents results for demographic variables, COVID-19 severity, and number of days between positive infection and laboratory testing in patients who were COVID^+^. Disease severity was mild and moderate, respectively, in 11.3% and 85.9% of patients who were COVID^+^, with no cases of severe disease. On average, participant testing occurred 179 ± 201 (SD) days after diagnosis in COVID^+^ (median, 84 days; interquartile range, 283.6 days). A total of four of the 71 patients in the COVID^+^ group reported that they were currently using tobacco.

**Table 1:** Descriptive characteristics and pulmonary function outcomes in COVID^−^and COVID^+^ participants.

Variable	COVID^−^, *n* = 55	COVID^+^, *n* = 71
**Demographic**		
Male/Female	24/31	21/50
Age, years	24 (21-30)	21 (20-25)^a^
Height, cm	168 (161-180)	168 (163-178)
Weight, kg	66.7 (56.7-80.3)	63.6 (56.8-73.2)
BMI, kg/m^2^	22.4 (20.9-25.2)	23.0 (21.4-25.3)
**Race**		
White/Hispanic/Asian/Not declared	48/0/7/0	57/3/9/2
**Severity**		
Asymptomatic/Mild/Moderate/Severe	NA	2/8/61/0
**Days between infection and lab testing**		
5-30		15
31-50		10
51-70		4
71-90		6
91-110		6
110-150		3
151-250		3
251-350		4
351-450		5
451-550		6
551-650		3
799		1
Not reported		5
**Spirometry**		
FVC, percent normal	103.3 ± 12.3	104.5 ± 12.3
FEV_1_, percent normal	100.3 ± 11.6	98.1 ± 10.9
FEV_1_/FVC, percent normal	96.9 ± 6.7	93.6 ± 8.3^a^
FEF_25-75%_, percent normal	94.5 ± 21.1	86.0 ± 21.2^a^
**Oscillometry**		
R5, percent normal	131.4 (39.8)	118.5 (52.7)
R5-R20, percent normal	206.5 (286.3)	262.5 (229.4)
X5, percent normal	133.7 (74.9)	127.3 (47.9)
AX, percent normal	182.4 (274.5)	234.8 (221.7)
**DL_CO_, percent normal**	113.1 ± 22.6	118.5 ± 18.2

DL_CO_, diffusing capacity for carbon monoxide; FEV_1_, forced expiratory volume 1 sec; FEF_25-75%_, forced expiratory flow between 25 and 75% FVC; FVC, forced vital capacity; R5, respiratory resistance at 5 Hz; R5-20, difference in respiratory resistance between 5 and 20 Hz; X5, reactance at 5 Hz; AX, area of the reactance curve between X5 and the resonant frequency.

Demographic variables presented as median with interquartile range in parentheses.

Race, severity, days between infection and testing presented as absolute number.

Spirometry data and DL_CO_ presented as mean ± SD.

Oscillometry data presented as median with interquartile range in parentheses

a *P* <0.05 vs COVID^−^.

**Figure 1a-e:**
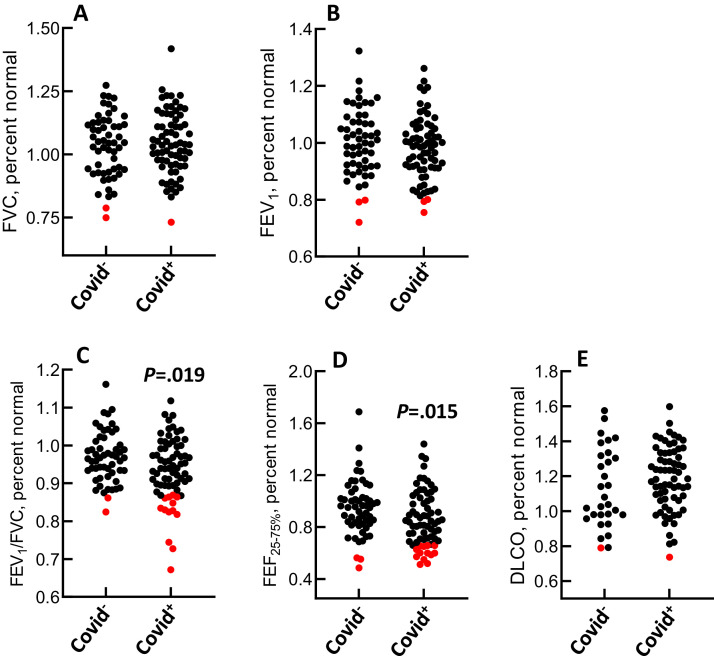
(a) FVC, (b) FEV_1_, (c) FEV_1_/FVC, (d) FEF_25-75%_, (e) DL_CO_ in patients who were COVID^−^ and COVID^+^. Each data point represents one patient. Red symbols indicate that the value is below the lower limit of normal. DL_CO_, lung diffusing capacity for carbon monoxide; FEV_1_, forced expiratory volume in 1 second; FEF_25-75%_, forced expiratory flow between 25 and 75% FVC; FVC, forced vital capacity.

The results for spirometric, oscillometric, and DL_CO_ measures are shown in Figure 1a-e and Table 1. FEV_1_/FVC was lower in participants who were COVID^+^ than those who were COVID^−^ (93.6 ± 8.3% vs 96.9 ± 6.7%pred [SD] in COVID^+^ vs COVID^−^, *P* = 0.019). FEV_1_/FVC was below the LLN in 13 patients who were COVID^+^ (18.3% of the group), whereas it was below the LLN in two participants who were COVID^−^ (3.6% of the group). FEF_25-75%_ was lower in participants who were COVID^+^ than those who were COVID^−^ (86.0 ± 21.2% vs 94.5 ± 21.1%-pred [SD] in COVID^+^ vs COVID^−^, *P* = 0.015). FEF_25-75%_ was below the LLN in 13 participants who were COVID^+^ and three patients who were COVID^−^. The chi-square analyses revealed significant associations between COVID-19 status and the number of values below LLN for FEV_1_/FVC (*P* = 0.012) and FEF_25-75%_ (*P* = 0.032). We tested for outliers using previously published methods [10]. None of the values for FEV_1_/FVC or FEF_25-75%_ were identified as outliers. None of the oscillometric variables were different between participants who were COVID^+^ and those who were COVID^−^. The number of days between COVID-19 infection and laboratory testing was not associated with any spirometric, oscillometric, or DL_CO_ outcome measure (data not shown).

## Discussion

We assessed pulmonary function in a group of young, healthy, mostly non-smoking, adults after recovery from COVID-19 and in a group who had never tested positive for COVID-19. Spirometry was mildly reduced in patients who were COVID^+^ compared with those who were COVID^−^; FEV_1_/FVC and FEF_25-75%_ were lower in the COVID^+^ group than the COVID^−^ group. However, neither DL_CO_ nor any of the oscillometric measures were different between the two groups. To the best of our knowledge, this study is the first to combine spirometry, forced oscillometry technique, and DLco data in a large cohort of mostly non-smoking men and women with and without COVID-19 infection. These findings provide new insights that persistent respiratory impairment after recovery from mild-to-moderate COVID-19 in young adults is rare and, if present, is mostly minor.

To the best of our knowledge, the range of days separating COVID-19 and testing in our cohort is the largest published to date. The number of days between active infection and pulmonary function testing in previous publications varies from a low of 1-2 weeks to a maximum of 6 months [2]. In other reports, the time interval between infection and testing was unclear or not stated [11]. If COVID-19 does cause lingering reductions in pulmonary function in young, healthy adults, it is reasonable to postulate that it would be most apparent in the early days after recovery and then progressively resolve over time. Our finding that there were no associations between the outcome measures and number of days separating infection and testing suggests that most young adults recover from COVID-19 without persistent decreases in pulmonary function.

The designs and methods used in previous reports on the long-term pulmonary outcomes of COVID-19 in young healthy adults are heterogeneous (e.g. differences in patient selection and age ranges, number of days separating infection and testing, disease severity, with or without vaccination), which limits the ability to integrate our findings with previous publications. Generally, others report either preserved or slightly compromised spirometry and DL_CO_ after COVID-19 in young, healthy adults [[2], [3], [4], [5], [6],^11^]. We note that sample sizes were very small in several of the studies reporting pulmonary function after COVID-19 [[2], [3], [4]]. The number of patients who were COVID^+^ in these three studies was between 18 and 29 persons. In one of the largest studies to date, Mogensen *et al.* reported unchanged spirometry from before to after the COVID-19 pandemic in 607 young adults who were seropositive for COVID-19 [11]. In another large study of young, healthy adults, Widmann *et al.* reported no difference in spirometry between a group of athletes who tested positive for COVID-19 and a group of control participants [5].

We do not know whether immunization status had an impact on our findings. Although we know that most of our participants had been vaccinated at the time of study, we do not know the proportion that were vaccinated before vs after infection. However, one can argue that the variable vaccination status among participants improves the generalizability of the findings.

## Conclusion

Two measures of forced expiratory airflow (FEV_1_/FVC and FEF_25-75%_) were mildly reduced in a group of young, healthy adults after COVID-19 infection compared with a group who had never tested positive for COVID-19. However, the reduction in the two variables was small, and neither oscillometric measures nor DL_CO_ were different between participants who were COVID^−^ and COVID^+^. Viewed collectively with previous findings, the evidence suggests that pulmonary function is largely preserved after mild-to-moderate COVID-19 in young, healthy adults.

## Declarations of competing interest

The author have no competing interests to declare.
